# Viral lysis modifies seasonal phytoplankton dynamics and carbon flow in the Southern Ocean

**DOI:** 10.1038/s41396-021-01033-6

**Published:** 2021-06-21

**Authors:** Tristan E. G. Biggs, Jef Huisman, Corina P. D. Brussaard

**Affiliations:** 1 Department of Marine Microbiology and Biogeochemistry, NIOZ Royal Netherlands Institute for Sea Research, Texel, The Netherlands; 2 Department of Freshwater and Marine Ecology, Institute for Biodiversity and Ecosystem Dynamics (IBED), University of Amsterdam, Amsterdam, The Netherlands

**Keywords:** Microbial ecology, Virus-host interactions

## Abstract

Phytoplankton form the base of marine food webs and are a primary means for carbon export in the Southern Ocean, a key area for global pCO_2_ drawdown. Viral lysis and grazing have very different effects on microbial community dynamics and carbon export, yet, very little is known about the relative magnitude and ecological impact of viral lysis on natural phytoplankton communities, especially in Antarctic waters. Here, we report on the temporal dynamics and relative importance of viral lysis rates, in comparison to grazing, for Antarctic nano- and pico-sized phytoplankton of varied taxonomy and size over a full productive season. Our results show that viral lysis was a major loss factor throughout the season, responsible for roughly half (58%) of seasonal phytoplankton carbon losses. Viral lysis appeared critically important for explaining temporal dynamics and for obtaining a complete seasonal mass balance of Antarctic phytoplankton. Group-specific responses indicated a negative correlation between grazing and viral losses in *Phaeocystis* and picoeukaryotes, while for other phytoplankton groups losses were more evenly spread throughout the season. Cryptophyte mortality was dominated by viral lysis, whereas small diatoms were mostly grazed. Larger diatoms dominated algal carbon flow and a single ‘lysis event’ directed >100% of daily carbon production away from higher trophic levels. This study highlights the need to consider viral lysis of key Antarctic phytoplankton for a better understanding of microbial community interactions and more accurate predictions of organic matter flux in this climate-sensitive region.

## Introduction

Phytoplankton blooms are the net result of gross growth minus losses [1] and the type of loss factor determines the flow of carbon and nutrients through the ecosystem [2]. Hence, studies that quantify viral lysis and grazing rates, and their functional significance throughout the productive season are greatly warranted. Zooplankton grazing is traditionally viewed as the dominant loss factor of phytoplankton cells [3], however, phytoplankton are also prone to viral infection [4–6]. Indeed, grazing rates do not always control primary production [7] and viral lysis may cause the collapse of phytoplankton populations [8, 9]. In contrast to grazing, viral lysis predominantly channels particulate organic carbon and nutrients away from higher trophic levels [2, 10]. The virus-induced release of host cellular content into surface waters fuels the so-called ‘viral shunt’ [11], whereby microbial processing of the released cell material directly affects biogeochemical cycling [12, 13]. All phytoplankton taxonomic groups are prone to infection by viruses [14–16] and the typically high host specificity of the lytic algal viruses can have a further regulatory influence on phytoplankton species succession [9, 17–19]. Although temporal variations in the relative magnitude of viral lysis and grazing could directly affect phytoplankton community composition and trophic transfer efficiency, studies on the seasonal dynamics of viral lysis rates in phytoplankton communities are scarce. Moreover, most knowledge on phytoplankton–virus interactions is from temperate oceans and coastal waters, whereas very little is known about the extent to which viral losses may affect phytoplankton populations in the cold, productive waters of the Antarctic [12, 20, 21].

The Southern Ocean is one of the largest carbon sinks of the global ocean [22, 23]. Its low temperatures are responsible for a relatively high solubility of carbon dioxide (CO_2_) and extensive phytoplankton blooms in the Southern Ocean absorb large amounts of CO_2_ for photosynthesis and growth [24, 25]. Primary production in Antarctic waters directs ecosystem productivity [26] sustaining large populations of copepods and krill [27] that in turn provide food for higher trophic levels (marine fish, birds and mammals) [26, 28, 29]. Portions of primary and secondary production become stored in the ocean interior by the biological carbon pump [30] and the formation of deep ocean water masses such as Antarctic Bottom Water and Antarctic Intermediate Water [31, 32]. Ocean warming and acidification due to anthropogenic activities have been predicted to impact Antarctic phytoplankton species composition, i.e. by shifting from larger diatoms to smaller-sized flagellates [33–36], which will directly impact food quality and availability for higher trophic levels in the Antarctic pelagic food web. If this shift in community composition coincides with changes in grazing and viral lysis rates, subsequent changes in the flux of organic matter and energy can be expected, with implications for the biological carbon pump. A detailed seasonal study is therefore required for a better understanding of the ecological role of viral lysis for Antarctic phytoplankton dynamics.

Here we report the seasonal dynamics of viral lysis rates of Antarctic phytoplankton groups, including picoeukaryotes, the prymnesiophyte *Phaeocystis* spp., cryptophytes and nano-sized diatoms. Our results reveal that viruses not only exert a major control over the population dynamics of these key phytoplankton groups, but that viral lysis is also critically important for obtaining a complete seasonal mass balance. These findings call for a reconsideration of the microbial food web and the efficiency of the biological carbon pump in this climate-sensitive region.

## Materials and methods

### Sampling site and procedure

This study was conducted at the Rothera time series site (RaTS, latitude 67.572°S; longitude 68.231°W [37]; located in Ryder Bay on the Western Antarctic Peninsula (Fig. S1). Samples were taken over a productive season from December 2012 to March 2013. Discrete seawater samples were collected from 15 m depth by a 12 L Niskin bottle deployed from a small boat. Full water column profiles were obtained using a SeaBird 19+ conductivity, temperature, depth instrument supplemented with a LiCor photosynthetically available radiation and an in-line fluorescence sensor (WetLabs). Sampling was conducted approximately once per week and processed directly upon return to the research base in a temperature-controlled lab (~0.5–1 °C).

### Phytoplankton

Seawater samples (1–8 L) were analysed for Chlorophyll *a* (Chl-*a*) concentration and for other pigments to determine taxonomic composition using high performance liquid chromatography (HPLC; see ref. [36] for details).

For phytoplankton enumeration (of single cells), 3.5 mL sub-samples were fixed with 100 µL formaldehyde-hexamine (18% v/v:10% w/v) at 4 °C for 15–30 min, after which they were snap-frozen in liquid nitrogen and stored at −80 °C until analysis. Samples were analysed according to [38] using a Becton Dickinson FACSCalibur flow cytometer equipped with an air-cooled Argon laser with an excitation wavelength of 488 nm (15 mW) and the trigger was set on red fluorescence. Phytoplankton populations (≤20 µm) were distinguished using bivariate scatter plots of red Chl-*a* auto-fluorescence versus side scatter. Cryptophytes were discriminated based on their orange phycoerythrin auto-fluorescence [39]. The flow cytometry data files were analysed using the freeware CYTOWIN [40]. No indications of chains or colonies were found in the cytograms and no significant difference was found in phytoplankton population counts between live and fixed samples (Mann–Whitney Rank Sum Test: *n* = 44 and *p* = 0.24).

Over the course of the season (and also using flow cytometry data from a consecutive season) ten phytoplankton populations could be distinguished, Phyto I–X, with average cell diameters of 0.9, 1.8, 3.1, 4.0, 4.5, 4.5, 7.4, 8.1, 11.5 and 20.4 µm (Fig. S2) [36]. The Phyto III population was identified as *Phaeocystis* spp. based on microscopic identification and resembling temporal dynamics with Prymnesiophyceae Chl-*a* as measured by HPLC [36]. Phyto IV were identified as cryptophytes based on flow cytometry counts and phycoerythrin orange auto-fluorescence, microscopic identification and matching temporal dynamics with Cryptophyceae Chl-*a* < 20 µm measured by HPLC. Phyto V-X were identified as diatoms based on microscopic confirmation of different diatom size classes and, at times they dominated, showed good comparisons between flow cytometry carbon and the cellular carbon of the related diatom Chl-*a* size fractions (<5, 5–20 and >20 µm, measured by HPLC after size fractionation) [36]. The standing stock of Phyto VII and X were consistently low (<50 mL^−1^) and were not included for rate analysis.

### Zooplankton, bacteria and viruses

Larger-sized zooplankton (>200 µm) were collected from surface waters (200–0 m) using a 200 µm mesh ring net (0.26 m^2^ opening) deployed from a small boat. Zooplankton samples were transported back to the laboratory in a portable cooler, preserved in 200 mL formaldehyde (5% final concentration) and stored at 4 °C until abundances were determined using a binocular microscope. For more detailed methodology see Biggs et al. [41].

Samples for bacteria and viruses were fixed (0.5% final concentration EM-grade glutaraldehyde; Sigma-Aldrich, Zwijndrecht, The Netherlands), stored at −80 °C after being flash-frozen in liquid nitrogen, and counted after thawing using flow cytometry according to the protocol by Marie et al. [42] and by Mojica et al. [43], respectively. In short, thawed samples were diluted in TE buffer (10 mM Tris HCl, 1 mM EDTA; pH 8.2) and stained with the nucleic acid-specific green fluorescent stain SYBR Green-I (Life Technologies, Bleiswijk, The Netherlands) to a final concentration of 1 and 0.5 × 10^−4^ of the commercial stock at room temperature (15 min) and 80 °C (10 min, after which the samples were cooled at room temperature in the dark for 5 min) for bacteria and viruses, respectively. For flow cytometric counts, the trigger was set on green fluorescence with the bacteria and different virus populations distinguished based on their green fluorescence and side scatter signal (Fig. S3).

Five distinct virus populations (V1–V5) were identified based on their green fluorescence and side scatter signal (Fig. S3). The commonly observed marine virus populations V1 and V2 are considered to be dominated by bacteriophages, although eukaryotic algal viruses (e.g. small genome-sized *Heterosigma* or diatom viruses) can also display similar low fluorescence signatures [14, 44–46]. The V3–V5 clusters generally contain more algal viruses, with prokaryotic and eukaryotic algal viruses contributing to the V3 group, and the V4 and V5 groups generally containing larger double-stranded DNA algal viruses [4, 47–49].

### Growth and mortality rates

To determine viral lysis and grazing rates of the identified phytoplankton populations, the predation pressures by smaller zooplankton (<200 µm; such as tintinnids, ciliates, heterotrophic dinoflagellates and nanoflagellates) and viruses were experimentally manipulated using the modified dilution assay [6]. In short, natural seawater was gently passed through a 200 μm mesh to remove larger-sized zooplankton such as large copepods and krill, and was then combined (by siphoning into 1L clear polycarbonate bottles) with either 0.45 μm (grazer-free) or 30 kDa (grazer and virus-free) filtered seawater at four dilutions (100, 70, 40 and 20% whole water), in triplicate. The 0.45 μm filtrate was prepared by gravity filtration of natural seawater through a 0.45 μm Sartopore capsule filter with a 0.8 μm pre-filter (Sartopore 2300, Sartorius Stedim Biotech, Göttingen, Germany). The 30 kDa ultra-filtrate was prepared by tangential flow filtration using a polyethersulfone membrane (Vivaflow 200, Sartorius Stedim Biotech, Göttingen, Germany). The dilutions were transferred in the dark to an outdoor flow-through incubator and incubated on a slow turning wheel (~0.5 rpm) at in situ temperature and light conditions (using neutral-density screens) for 24 h. Phytoplankton were enumerated at the start and end of the incubation using flow cytometry (see above) and the apparent growth rate was calculated based on the natural logarithm. By using flow cytometry, the loss rates could be specified for each phytoplankton group. The (negative) slopes of linear regressions of the apparent growth rate versus the actual dilution factor provides both specific grazing (0.45 μm series) and grazing + viral lysis (30 kDa series) rates with viral lysis rates derived by subtraction [50]. In those cases where the slope of the linear regression was positive and >0.1, we considered that proper estimation of the grazing and viral lysis rates failed and the values were discarded [50]. In the case of missing replicates (e.g. due to technical error during the assay or flow cytometric analysis) or clear outliers (compared to the series, often when population abundance was low), we performed the regression analysis without those values. Actual dilution factors were determined by dividing the total phytoplankton count of the specific dilution (of the 0.45 µm or 30 kDa series) by the average total count of the 100% natural water. The y-axis intercept of the linear regression represents the gross specific growth rate (*µ*_*gross*_), assuming the sum of the specific grazing and viral lysis rates of these phytoplankton (≤20 μm) to equal the total specific mortality rate (*m*_*tot*_).

### Carbon production and losses

To calculate daily amounts of carbon produced by phytoplankton production and lost by grazing and viral lysis, we assume that the population dynamics of the phytoplankton can be described by a simple model:

1 $$\frac{{dX}}{{dt}} \,=\, \left( {\mu _{gross} \,-\, m_{tot}} \right)X$$

where *X* is the phytoplankton concentration at time *t*, *µ*_*gross*_ is the gross specific growth rate, and *m*_*tot*_ is the total specific mortality rate driven by viral lysis and grazing (i.e. *m*_*tot*_ = *m*_*V*_ + *m*_*G*_).

Assuming that the specific growth and mortality rates do not change over sufficiently short time intervals, this differential equation can be solved as:

2 $$X(t) \,=\, X_0\,e^{\left( {\mu _{gross} \,-\, m_{tot}} \right)t}$$

The total number of phytoplankton cells produced over a time interval of *T* days (*P*_*T*_) can be calculated by integrating the production rate over time:

3 $$P_T \,=\, \int_{0}^{T} \mu _{gross}X(t)dt$$

Inserting Eq. (2) into Eq. (3) and subsequent integration gives:

4 $$P_T \,=\, \frac{{\mu _{gross}}}{{\mu _{gross} \,-\, m_{tot}}}\left( {X_0\,e^{\left( {\mu _{gross} \,-\, m_{tot}} \right)T} \,-\, X_0} \right)$$

Similarly, the total number of phytoplankton cells lost over *T* days (*L*_*T*_) can be calculated as:

5 $$L_T \,=\, \frac{{m_{tot}}}{{\mu _{gross} \,-\, m_{tot}}}\left( {X_0\,e^{\left( {\mu _{gross} \,-\, m_{tot}} \right)T} \,-\, X_0} \right)$$

This total loss is partly due to viruses and partly to grazers. Specifically, the total number of phytoplankton cells lost over *T* days by viral lysis is (*m*_*V*_/*m*_*tot*_)*L*_*T*_ and by grazing is (*m*_*G*_/*m*_*tot*_)*L*_*T*_. We calculated daily rates, with a time interval of *T*  = 1 day, in-line with the 24 h incubation period used in the dilution assays.

To convert cell numbers to carbon, the total number of cells produced and lost (i.e. *P*_*T*_ and *L*_*T*_) were multiplied with the cellular C content (µg C cell^−1^) of each phytoplankton group. The cellular C content of each phytoplankton population was estimated from the average cell diameter, using conversion factors of 237 and 196.5 fg C µm^−3^ for pico-sized (Phyto I–III) and nano-sized (Phyto IV–IX) phytoplankton populations, respectively [51, 52]. To calculate total seasonal production and loss rates, we integrated daily carbon produced (µ_gross_), grazed, lysed and lost (*m*_*tot*_) over the entire length of our sampling period (from 6 December 2012 to 12 March 2013) with linear interpolation between data points using R statistical software (R Development Core Team 2012).

### Statistics

According to the modified dilution assay [4, 6], a significant difference from zero for the grazing regression coefficient (0.45 µm series) indicates significant grazing rates, whilst a significant difference between the regression coefficients of the 0.45 µm and 30 kDa series (as tested by analysis of covariance, *p* < 0.05, using R statistical software; R Development Core Team 2012) indicates significant viral lysis rates. A significant difference from zero for the regression coefficients of the 30 kDa series indicates significant gross growth. Throughout the text the plus–minus symbol (±) represents 1 standard deviation. Significant differences between viral lysis and grazing rates were tested using a two-tailed Student’s *t* test and a Mann–Whitney *U* test if data deviated from normality as assessed by the Shapiro–Wilk test (SigmaPlot v14, from Systat Software, Inc., San Jose California, USA). The strength of the relationship between viral lysis and grazing rates was determined using Pearson’s correlation (SigmaPlot v14).

## Results and discussion

### Specific phytoplankton viral lysis rates

This study details the specific viral lysis rates for eight different Antarctic phytoplankton populations throughout the entire Austral productive season. To our knowledge, this is the first detailed viral lysis rate study of multiple phytoplankton species during major seasonal changes in plankton community composition and size distribution, not only for coastal waters of the Western Antarctic Peninsula (Fig. S1), but for the entire Southern Ocean.

**Fig. 1:**
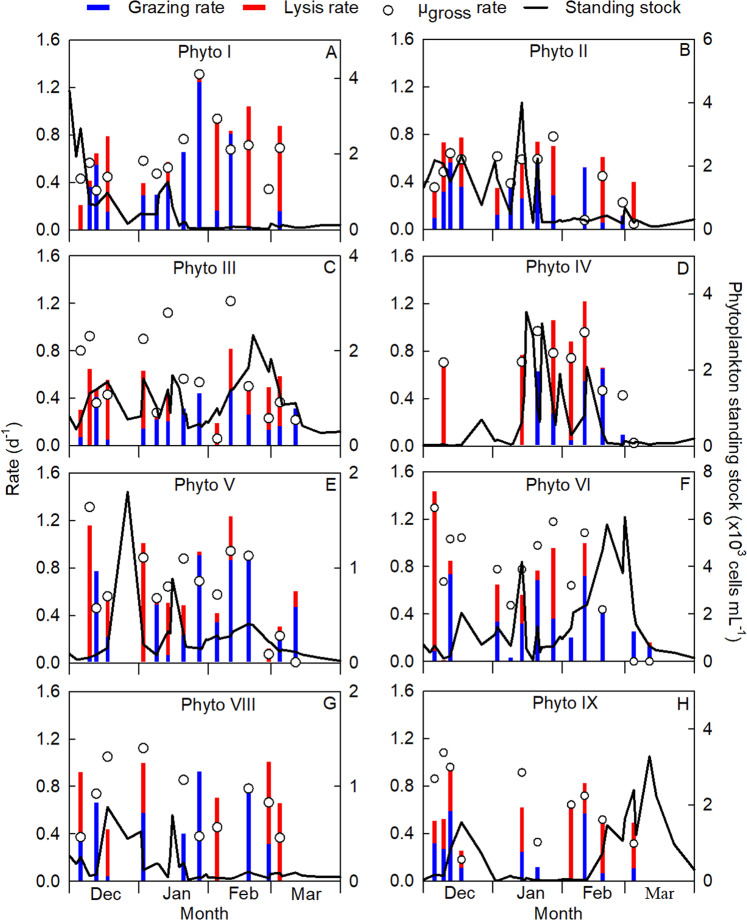
Seasonal dynamics of phytoplankton standing stock (green line), specific gross growth rate (µ_gross_; open circles), viral lysis rate (red bars) and grazing rate (blue bars) for different phytoplankton groups distinguished by flow cytometry. The phytoplankton groups include (**A**, **B**) picoeukaryotes (Phyto I and II), (**C**) *Phaeocystis* (Phyto III), (**D**) cryptophytes (Phyto IV), and (**E–H**) diatoms of different size classes (Phyto V, VI, VIII and IX). Note that the scale for standing stock (right *y*-axis) differs between the panels; standing stock data are composed of *n* = 41 data points.

Although light microscopy of Lugol’s fixed seawater samples revealed relatively high numbers of micro-sized centric diatoms in mid-December (measuring 25–60 µm; unpublished data), the pico- and nano-sized phytoplankton ≤20 µm contributed 70% (±22%, *n* = 13) to total Chl-*a* over the entire season and their contribution increased to 77% (±16%, *n* = 11) from late December until the end of the season (Fig. S4). The temporal dynamics of the different phytoplankton groups (Fig. 1) and total Chl-*a* concentration (Fig. S4) showed that at the start of the productive season (beginning of December) the smaller-sized Phyto I (average cell diameter of 0.9 µm), Phyto II (1.8 µm Ø), and *Phaeocystis antarctica* Phyto III (3.1 µm Ø) were numerically dominant (58%, 16% and 10%, respectively). Thereafter, the abundance of the diatom groups (Phyto V, VI, VIII and IX, 4.5–11.5 µm Ø) increased during the ‘spring’ bloom in the second half of December. The ‘spring’ bloom was followed by dominance of smaller phytoplankton, particularly cryptophytes Phyto IV (4 µm Ø), driven by the relatively warm austral summer months [36]. Later in the productive season (February) when temperature was still relatively high, salinity had become relatively low due to melting ice and light was not yet limiting, *Phaeocystis* Phyto III and diatoms Phyto VI bloomed (Fig. 1). Finally, they were succeeded by diatoms Phyto IX during late summer and austral fall (mid-February to March) when light availability and nutrient (phosphate and nitrate) concentrations declined as a result of shorter daylengths, increased light attenuation and biological nutrient uptake [36]. Although we did not specifically identify the species of diatoms during the season, van Leeuwe et al. [53] observed relatively high numbers of *Thalassiosira* sp. (5–20 µm cell length) and *Chaetoceros* sp. (10–15 µm cell length) at the same location in January and February.

Overall, specific viral lysis rates were substantial for all phytoplankton groups (Fig. 1 and Table S1), with average lysis rates ranging between 0.23 ± 0.38 (*n* = 13, Phyto VI) and 0.42 ± 0.39 d^−1^ (*n* = 9, Phyto IV). Comparatively high rates of viral lysis have previously been reported for picoeukaryotic phytoplankton (0.23–0.81 d^−1^) [54, 55], for *Phaeocystis globosa* (0.35 d^−1^) [4] and for nano-eukaryotes (up to 0.89 d^−1^) [6]. The virus populations V4 and V5 (Fig. S3) resembled the flow cytometric signatures of large genome dsDNA algal viruses such as *Phaeocystis* viruses [4, 49], whereby the temporal dynamics of V4 matched best with Phyto III (Fig. S5a). The occurrence of potential eukaryotic algal virus populations together with considerable viral lysis rates seems to give support to our findings, however, flow cytometric analysis alone cannot provide certainty. Virus population V5 corresponded best with diatom Phyto VI (Fig. S5b), despite that thus far no large genome dsDNA viruses infecting diatoms have been reported.

Although diatom populations are known to be infected by viruses [15, 56, 57], specific viral lysis rates for natural diatom populations have not yet been reported in the literature. Our results demonstrate that viral lysis of diatoms (i.e. Phyto V, VI, VIII and IX) can be substantial (Fig. 1E–H). They displayed a seasonally average viral lysis rate of 0.29 ± 0.32 d^−1^, which is almost of equal magnitude as their seasonally averaged grazing rate of 0.35 ± 0.27 d^−1^ (both *n* = 47; Table S1). Antarctic diatoms often dominate high-biomass phytoplankton blooms and are an important source of primary nutrition as they are major producers of long-chain polyunsaturated fatty acids (such as 20:5ω-3) [58]. The consumption of diatoms has been reported to increase the sedimentation speed of fecal pellets due to the ballasting effect of biogenic silica [59, 60]. Instead, viral lysis will promote the recycling of dying host cells in surface waters [61]. Recent research, however, has shown that viral lysis may have the potential to also induce aggregate formation and potentially sedimentation [62].

### Ecological and seasonal importance of viral lysis

Viral lysis rates varied over the season (Fig. 1A), with Phyto I displaying significantly higher grazing than lysis rates during the first half of the season (average 0.44 ± 0.36 d^−1^ and 0.15 ± 0.20 d^−1^, respectively; Mann–Whitney *U* test: *U* = 16, *n*_1_ = *n*_2_ = 9, *p* = 0.034). Nutrient depletion, as found at the end of December [36], could have negatively impacted virus production [63, 64], however, nutrient stress was brief due to mixing at the beginning of January. During the second half of the season (February and March), viral lysis appeared to be the dominant loss factor for Phyto I (0.52 ± 0.47 d^−1^ compared to grazing 0.23 ± 0.33 d^−1^, *n* = 5), which may have contributed to its reduced standing stock (Fig. 1A). During the course of the blooms of *Phaeocystis* Phyto III and cryptophytes Phyto IV, their viral lysis rates were substantial (0.30 ± 0.26 d^−1^ and 0.61 ± 0.35 d^−1^, respectively; Fig. 1C, D). In contrast to Phyto I, Phyto V experienced high losses by viral lysis during the first half of the season (0.45 ± 0.44 d^−1^, *n* = 7) whereas during the second half the share of grazing was significantly higher (0.53 ± 0.37 d^−1^ vs. lysis 0.12 ± 0.12 d^−1^; Student’s *t* test: *t*_12_ = 2.787, *n*_1_ = *n*_2_ = 7, *p* = 0.0164; Fig. 1E). Unlike Phyto III and IV, viral lysis rates were low for diatom Phyto VI (0.06 ± 0.12 d^−1^; Fig. 1F) when they reached high abundances in February and March. Similar to Phyto I in the second half of the season, high lysis rates were measured for diatoms Phyto VIII and IX (0.51 ± 0.34 d^−1^ and 0.42 ± 0.14 d^−1^, respectively; Fig. 1G, H). Temporally separated populations of phytoplankton may exhibit variability in the susceptibility to both viral lysis and grazing depending on the life history of both predator (viruses and grazers) and prey (phytoplankton).

**Fig. 2:**
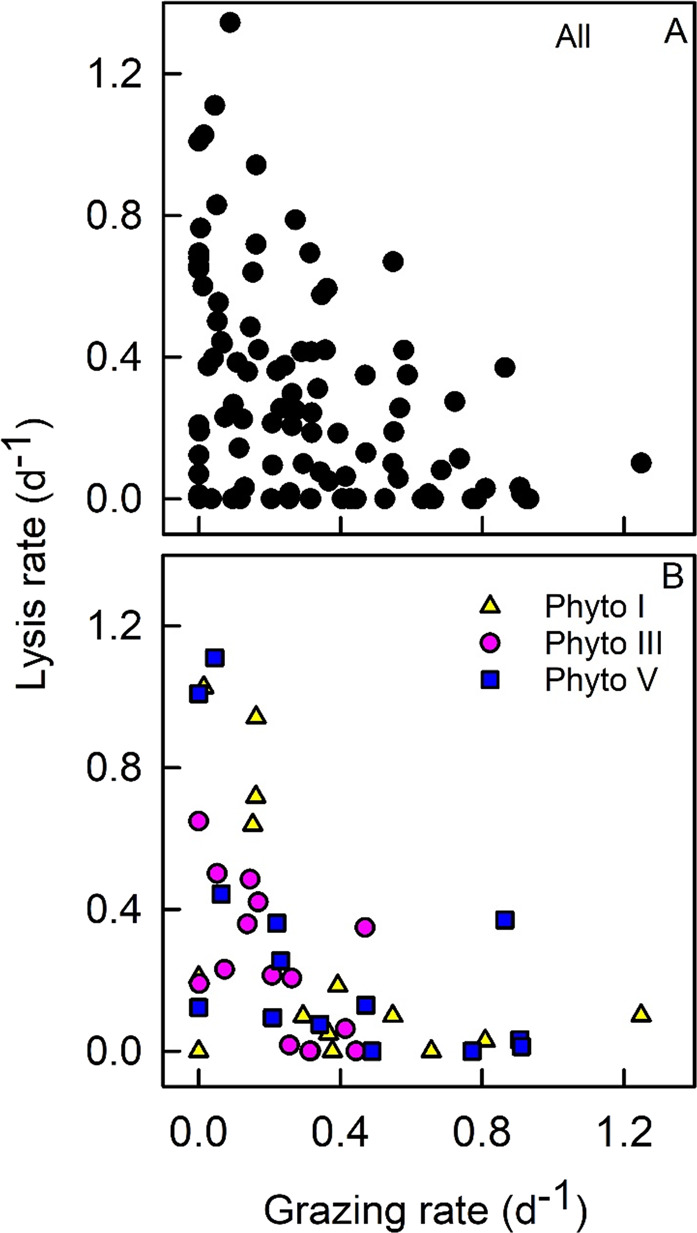
Correlation of viral lysis and grazing rates. Specific viral lysis rates are plotted against grazing rates for (**A**) all phytoplankton groups and (**B**) phytoplankton groups Phyto I, III and V only.

Our results show that high viral lysis rates coincide with low grazing rates and vice versa (Pearson’s *r* = −0.43, *n* = 98, *p* < 0.0001; Fig. 2A), in particular for picoeukaryote Phyto I, *Phaeocystis* Phyto III and diatom Phyto V (Fig. 2B, Table S2). A negative correlation between viral lysis and grazing could arise from physiological control by the algal host cell, where an increase in the length of the latent period could be favourable for losses by grazing (i.e. cells have a greater chance of being grazed before cell lysis), in contrast to shorter latent periods that might favour losses due to viral lysis [64–68]. A negative correlation may also arise from direct or indirect interactions among grazers and viruses, such as preferential grazing on or avoidance of virally infected phytoplankton cells [69, 70], or direct grazing of (algal) viruses, for example by heterotrophic nanoflagellates [71]. The impact of larger-sized (≥200 µm) zooplankton (Fig. S6) on smaller zooplankton (<200 µm) could also influence the balance between the phytoplankton loss factors.

**Fig. 3:**
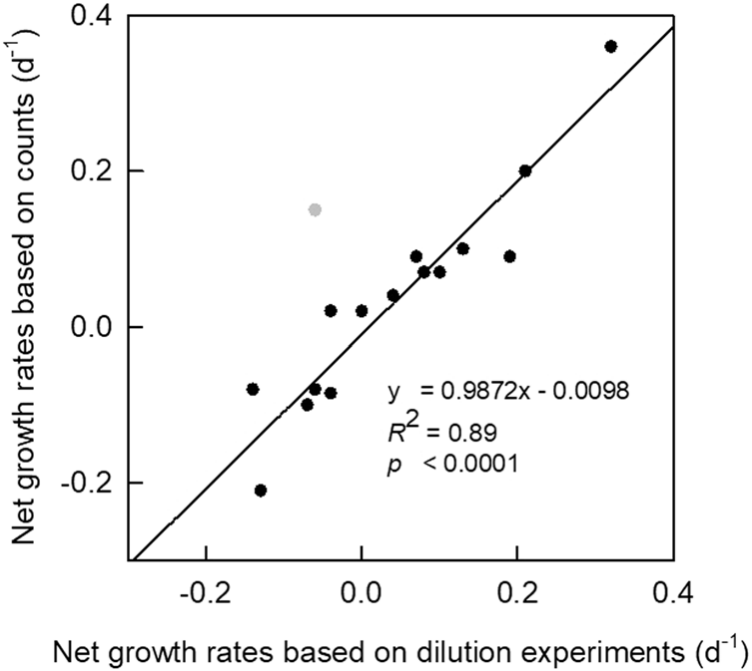
A comparison of net specific growth rates (µ_NET_) based on phytoplankton counts and on dilution experiments. Dilution experiment µ_NET_ rates were averaged (*n* ≥ 3) over time periods of sustained increase and decrease in population abundance and compared to µ_NET_ rates estimated from in situ abundance counts over the same time period. Note: outlier shown in grey is not included in the linear regression.

**Fig. 4:**
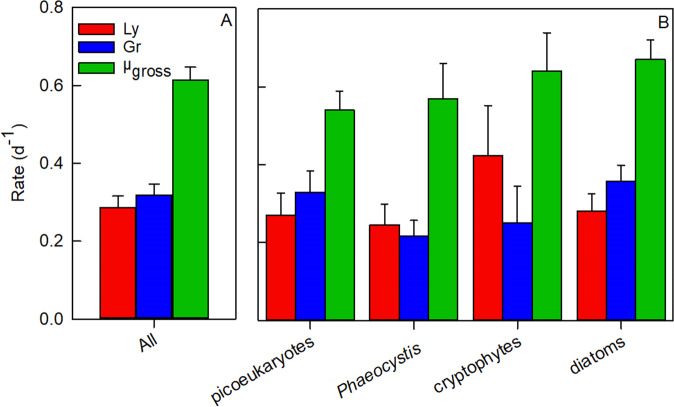
Seasonal mean specific rates (d^−1^) of viral lysis (Ly), grazing (Gr) and gross growth (µ_gross_). (**A**) All phytoplankton (All), and (**B**) per phytoplankton group: picoeukaryotes (Phyto I and II), *Phaeocystis* (Phyto III), cryptophytes (Phyto IV) and diatoms (Phyto V, VI, VIII and IX). Error bars represent ±1 standard error.

Net growth rates based on counts from the mortality assay (for distinct periods of abundance increase and decline, where experimental *n* ≥ 3) aligned well with net growth rates obtained from in situ abundance dynamics (Fig. 3), indicating that the experimental rates (Table S1) provided a very good representation of actual growth and loss processes. Averaged over the entire productive season, specific viral lysis rates were comparable to grazing rates (0.29 ± 0.30 d^−1^ and 0.31 ± 0.27 d^−1^, respectively, *n* = 98; Fig. 4A). The trend that viral lysis rates were of similar magnitude as grazing rates held for all major phytoplankton populations and taxonomic groups, i.e. picoeukaryotes, *Phaeocystis*, cryptophytes and diatoms (Fig. 4B). Furthermore, total mortality (sum of viral lysis and grazing) matched gross growth when averaged over the entire season (Fig. S7).

**Fig. 5:**
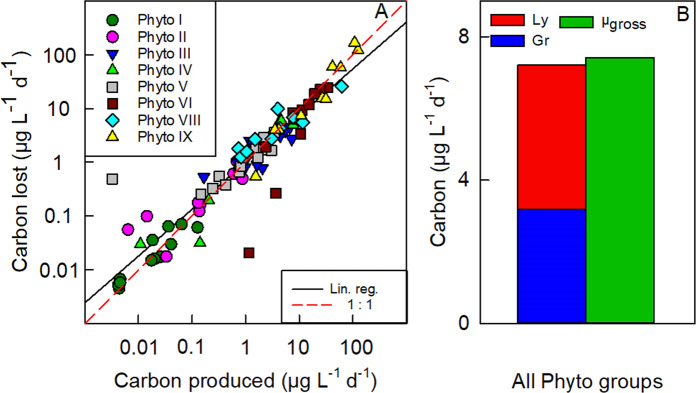
Seasonal phytoplankton carbon production and losses. **A** Daily phytoplankton carbon lost by viral lysis and grazing plotted against daily carbon produced by gross growth of the phytoplankton populations. Different data points are from rate measurements at different time points during the season. **B** Seasonal mean of total phytoplankton carbon lost by viral lysis (Ly) and grazing (Gr) and total carbon produced by gross growth, overall phytoplankton groups. The regression line (solid black line) in (**A**) has a slope of 0.87 (*R*^2^ = 0.87; *p* < 0.0001, *n* = 95). Note: zeros (*n* = 1) and negative (*n* = 2) values were excluded from log transformed carbon data in (**A**).

The ecological importance of viral lysis is highlighted by a close-to-zero mass balance of seasonal phytoplankton carbon production and losses when viruses are included as a loss factor (Fig. 5). Furthermore, differences in the slopes of the individual linear regressions (Fig. S8) suggest there were differences in the relation between carbon production and carbon losses among phytoplankton groups. The extent to which grazers and viruses can control different phytoplankton populations depends on multiple factors such as size class and abundance of phytoplankton, grazers and viruses, nutrient and light availability, susceptibility to grazing and viral lysis at each moment in time, and the ability of grazer and virus populations to keep up with increasing phytoplankton production [72]. Our data demonstrate that viral lysis is not only an important cause of mortality for Antarctic phytoplankton (of equal magnitude as grazing by smaller zooplankton) but also an essential process to be included for a better understanding of phytoplankton population dynamics. Relatively high growth rates and comparably high total loss rates (grazing plus lysis) of the dominant Antarctic phytoplankton (≤20 µm fraction, typically ruling total Chlorophyll *a*; Fig. S4) suggests that loss processes are coupled to growth and indicate a rapid turnover of photosynthetically fixed carbon that helps to explain the relatively low concentration and slow build-up of biomass in comparison to available resources.

### Carbon flux and potential future implications

Overall, viral lysis was a major loss factor responsible for a seasonal carbon flux of 2674 µg C L^−1^, which represented 58% of the total seasonal phytoplankton carbon losses (4645 µg C L^−1^) and 55% of the total seasonal phytoplankton carbon produced (4821 µg C L^−1^). The nano-sized diatoms accounted for the majority of the total cellular carbon grazed, lysed and produced (91%, 91% and 91%, respectively), whereby Phyto IX (average cell diameter 11.5 µm) contributed most (55%, 71% and 56%, respectively; data not shown). In contrast to smaller nano-sized diatoms such as Phyto V-VIII, larger nano-sized diatoms such as Phyto IX are considered a suitable prey size for larger zooplankton such as copepods [73–75]. We did not estimate grazing rates by zooplankton >200 µm, and hence we do not know the full extent to which Phyto IX was grazed. However, during the December spring bloom, the net growth rates of Phyto IX based on natural abundances (0.20 d^−1^) matched the average net growth rates of Phyto IX from the dilution experiments (mean = 0.21 d^−1^, *n* = 4), suggesting the majority of losses were accounted for by the experimental incubations. Accordingly, losses by viral lysis of Phyto IX during the December experiments (spring bloom period) were most likely considerable (Table S3) as they diverted an estimated 30% of total diatom carbon produced (91 µg C L^−1^) away from higher trophic levels. Additionally, during the late summer bloom (dominated by Phyto IX, Fig. 1H) viral lysis can at least occasionally direct the vast majority of daily carbon produced towards the dissolved pool. For example, during the large ‘lysis event’ on 5 March, 124% (135.5 µg C L^−1^ d^−1^) of daily carbon produced (109.4 µg C L^−1^ d^−1^) was lost by viral lysis (Table S3). Such a considerable flux of organic matter from the particulate to dissolved fraction (viral shunt) stimulates bacterial production [10, 76] and hence may explain the strong increase in bacterial abundance at the end of the season (0.1–2.4 × 10^6^ cells mL^−1^ from mid-February to mid-March, Fig. S9). Virus mortality pressure may have been responsible for the subsequent decline in bacteria, as suggested by the peaks in total virus abundance in the second half of March and early April (Fig. S9). The substantial viral lysis of Phyto IX could have reduced phytoplankton food availability for key feeding stages of herbivorous copepods and may have impacted lipid accumulation (and annual reproductive success) at a crucial time prior to overwintering [41].

Climate change scenarios predict enhanced sea-ice loss in Antarctic waters [77] that is likely to increase the length of the productive period [78, 79]. However, earlier and strengthened stratification (due to higher temperatures and ice melt) [80–82] will reduce the flux of dissolved inorganic nutrients to surface waters [83] and is predicted to promote a more flagellate-dominated community (cryptophytes Phyto IV, *Phaeocystis* Phyto III) with less larger-sized diatoms during the summer months [36, 84–86]. Our study shows that viral lysis was a major loss factor for cryptophytes and *Phaeocystis* (Fig. 4B). The share of viral lysis as compared to grazing is therefore expected to increase, even if these flagellates become more dominant. Moreover, ocean acidification has been shown to diminish diatom silicification [33] and if this results in silicate stress for the (smaller-sized) diatom cells, viral infection and mortality of diatoms could be enhanced [56]. Under these future scenarios, our study implies that viral lysis will play a more prominent role, reducing trophic level transfer efficiency and ecosystem productivity.

Our study demonstrates that viral lysis not only implements decisive control over the seasonal dynamics of the different ecologically important Antarctic phytoplankton groups (picoeukaryotes, the prymnesiophyte *Phaeocystis* spp., cryptophytes and diatoms), but also reveals how imperative it is to include viral lysis in mass-balance calculations. Mathematical models that involve Antarctic phytoplankton dynamics and seasonal mass balance, but do not account for viral lysis derived losses, are likely to overestimate the impact of grazing. Our findings highlight that viral lysis currently redirects about half (55%) of seasonal phytoplankton carbon production towards the microbial loop, indicating that carbon sequestration by these Antarctic ecosystems is less effective than previously believed. This necessitates a reconsideration of the efficiency of the biological pump in Antarctic waters.

## Supplementary information

Supplementary Information

## Data Availability

All data used in this publication, including the script used to test for significant differences between dilution series regressions; and a subset of representative flow cytometry files (consisting of four complete experiments from various time points throughout the season), are publicly available at https://dataverse.nioz.nl/dataset.xhtml?persistentId=doi:10.25850/nioz/7b.b.hc.
