# Supplementary material for: Viral lysis modifies seasonal phytoplankton dynamics and carbon flow in the Southern Ocean
Source: ISME J. 2021 Jun 21;15(12):3615–22. doi: 10.1038/s41396-021-01033-6 (PMC8630045; doi:10.1038/s41396-021-01033-6)
Supplement: Supplementary file 1 — Supplementary Information [file 41396_2021_1033_MOESM1_ESM.docx]

**Viral lysis modifies seasonal phytoplankton dynamics and carbon flow in the Southern Ocean**

**Supplementary Figures**


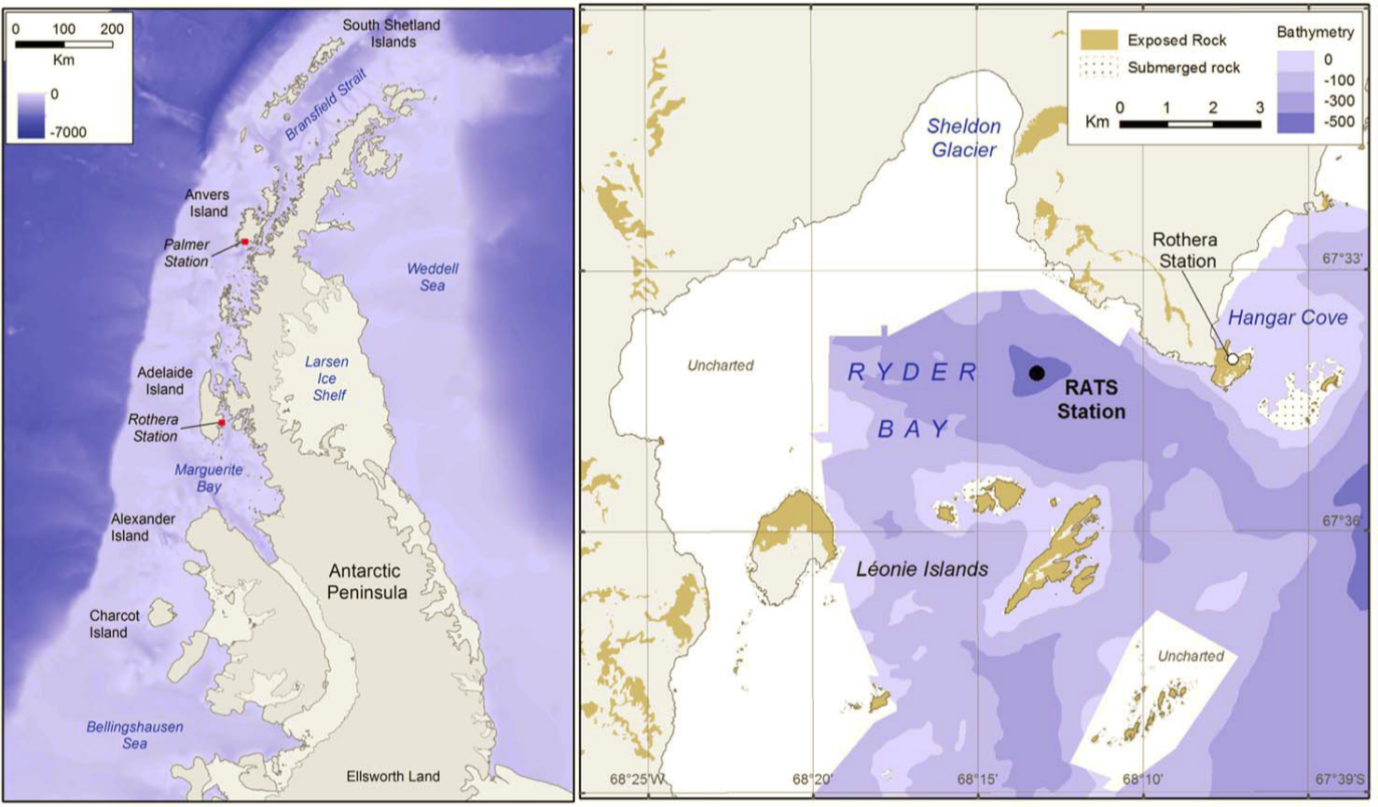


Fig. S1. Map of the study site: (a) the location of Rothera research station in northern Marguerite Bay. Note: Ryder Bay is to the east of Adelaide Island and to the west of the Antarctic Peninsula. (b) large scale map of the Rothera Time Series (RATS) sample site within Ryder Bay. Map adapted from Annett et al. 2015 [1].


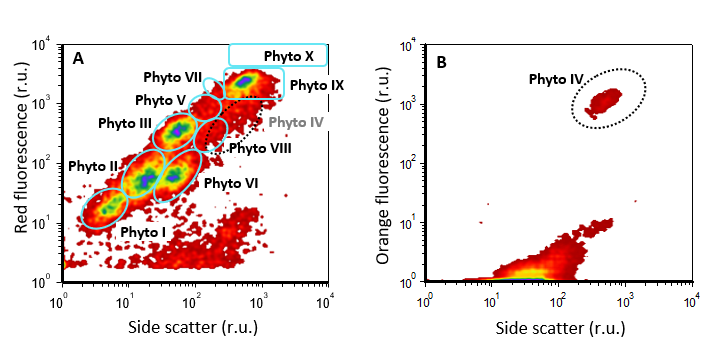


Fig. S2. Cytogram plots indicating the location of Phyto groups I to X. (A) All phytoplankton populations were discriminated (blue lines) based on chlorophyll red autofluorescence versus side scatter, except for Phyto IV (indicated with black dotted line). (B) Cryptophytes Phyto IV were discriminated based on orange autofluorescence versus side scatter. The location of Phyto VII and X were based on another consecutive season with cleared gates due to higher abundances of these groups in that season. For analysis, gates were connected to include all cells (cells in overlapping sections were corrected for). Axis are in relative units (r.u.). Note: Populations may shift over the season as a result of factors such as changes in light and nutrient availability (and occasionally flow cytometer settings change).


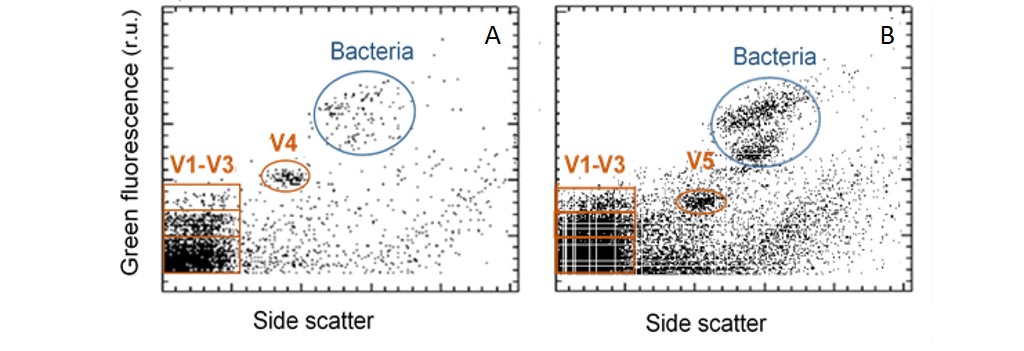


Fig. S3. Cytograms showing the location of the V4 (a) and V5 (b) virus groups, in relation to bacteria and smaller virus groups V1–V3.


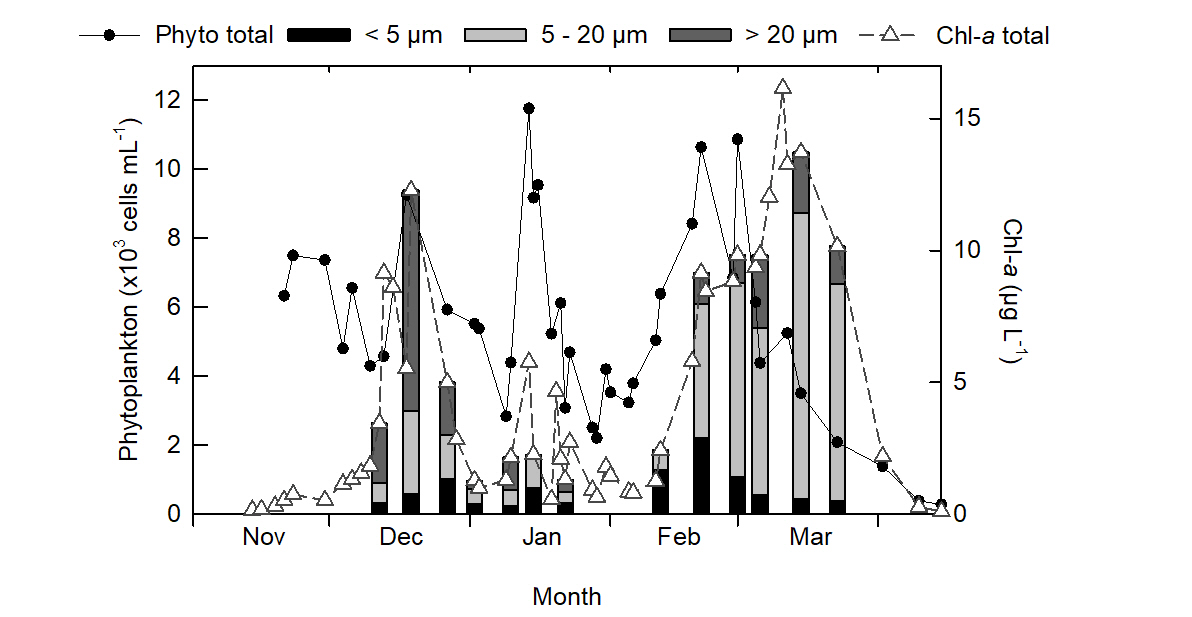


Fig. S4. Time series of total phytoplankton abundance as determined by flow cytometry (Phyto total; black circles and solid line) and total Chlorophyll-*a* concentrations as determined by high performance liquid chromatography (Chl-*a* total; open triangles and dashed line) at the RATS sample site (15 m depth) in Ryder Bay. Bar chart indicates the fraction of Chl-*a* total contained within three size classes (< 5, 5 – 20 and > 20 µm).


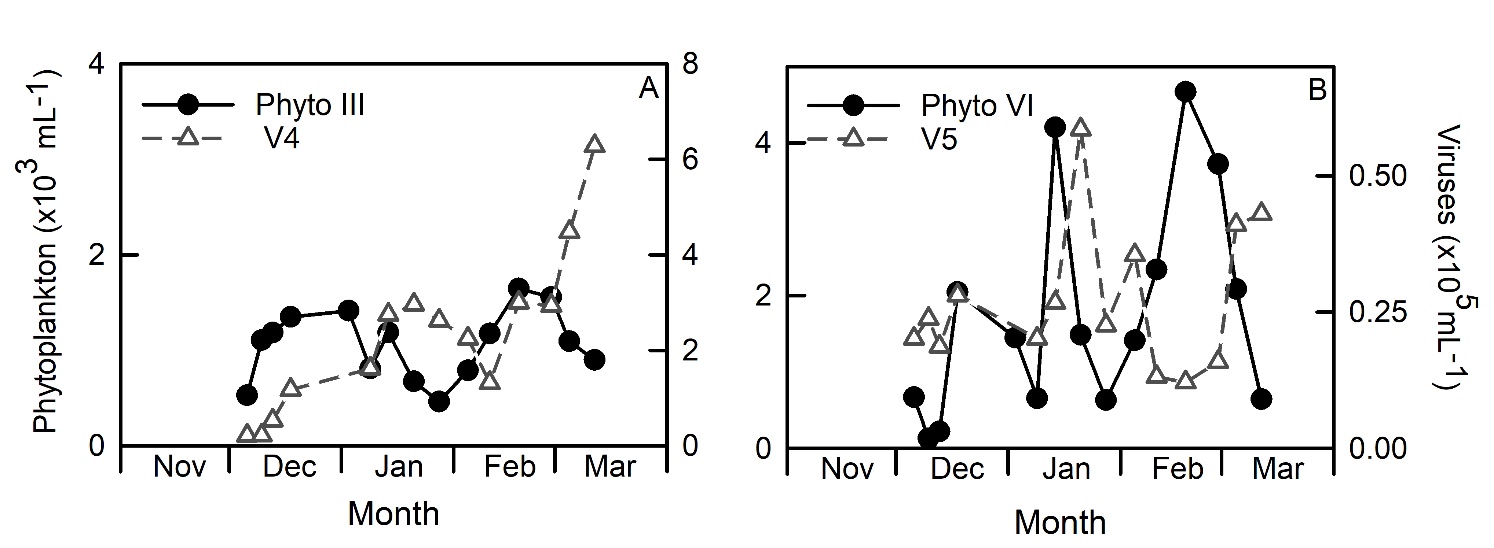


Fig. S5. Comparisons of the temporal dynamics of phytoplankton and virus counts on experimental days for (a) Phyto III vs virus group V4 and (b) Phyto VI vs virus group V5. Note the different scales between y-axes.


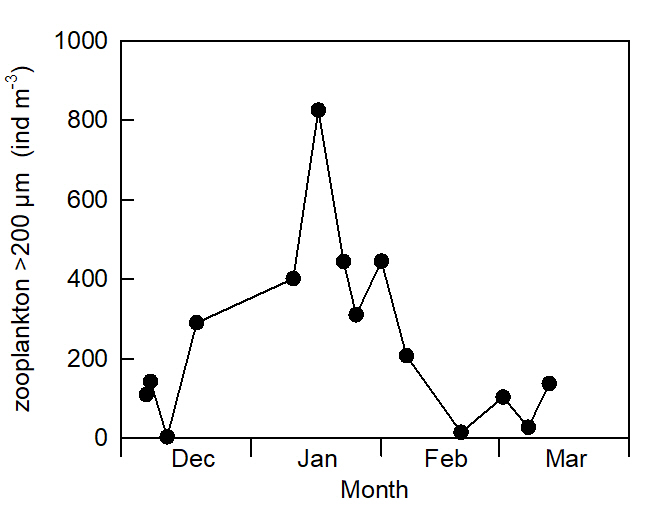


Fig. S6. Time series of larger-sized zooplankton abundance (≥ 200 µm) at the sample site from surface water (200 – 0 m) net hauls.


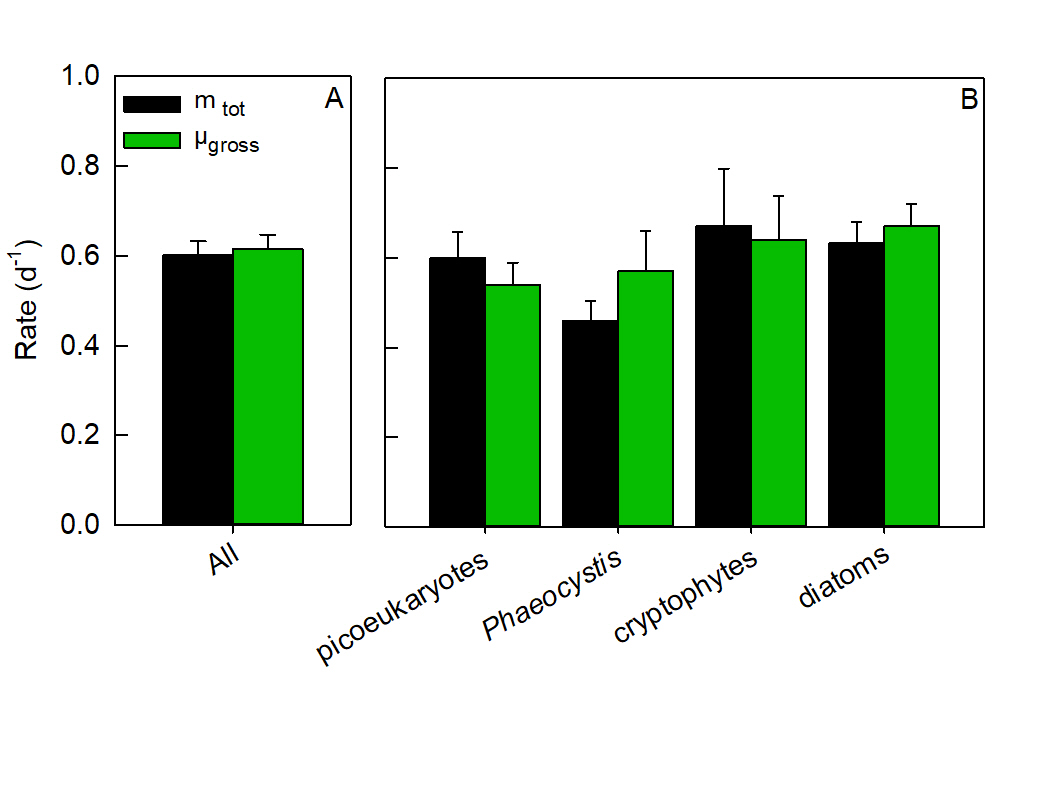


Fig. S7. Seasonal mean specific rates (d^-1^) of gross growth (µ_gross_) and total mortality (m_tot_, i.e. lysis plus grazing) are shown in (a) for all phytoplankton groups (All) and (b) per group: *pico-eukaryotes* Phyto I and II; *Phaeocystis* Phyto III, *cryptophytes* Phyto IV and *diatoms* Phyto V, VI, VIII and IX. Error bars represent ± 1 standard error.


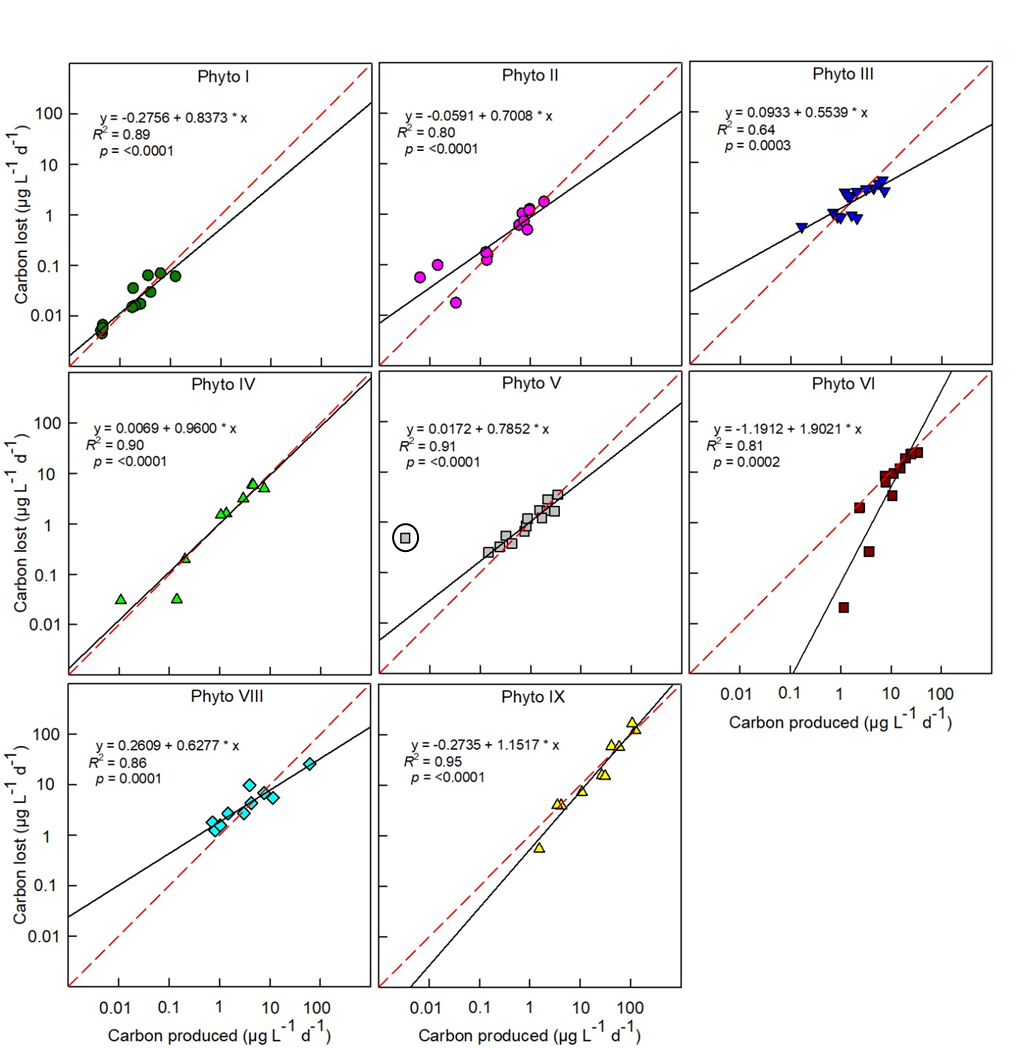


Fig. S8. Log transformed phytoplankton carbon produced by gross growth plotted against carbon lost by viral lysis and grazing, obtained after conversion of the different phytoplankton group abundances to carbon (calculated from the specific viral lysis, grazing, total mortality and gross growth rates (d^-1^)) for each phytoplankton group (Phyto I-VI and VIII-IX). Different data points are converted from rate measurements at different timepoints during the season. In each sub-plot the linear regression line (solid black), its formula and the 1:1 line (red dashed) are included. Note: zeros (*n* = 1 for Phyto I on 28/02/2013) and negative (*n* = 2 for Phyto VI on 05/03/2013 and 12/03/2013) values were excluded from log transformed carbon data. Also, the outlier circled in sub-plot Phyto V was excluded from the linear regression.


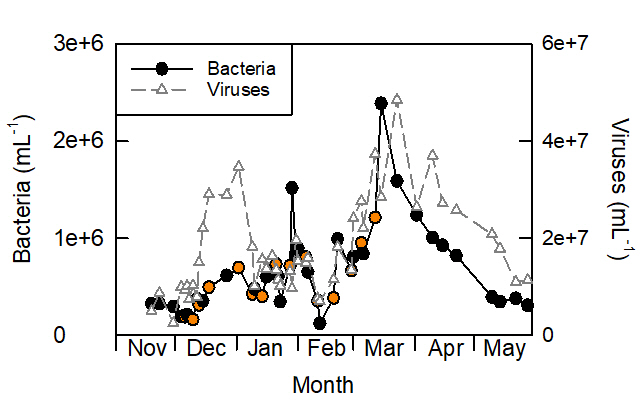


Fig. S9. The temporal dynamics of bacteria and total (V1-V5) virus numbers (mL^-1^). Orange (bacteria) datapoints represent experimental dates i.e. 6^th^, 10^th^, 13^th^ and 18^th^ December; 3^rd^, 9^th^, 14^th^, 21^st^ and 28^th^ January; 5^th^, 11^th^, 19^th^ and 28^th^ February; and 5^th^ and 12^th^ March.

**Supplementary Tables**

Table S1. Temporal variation in specific viral lysis (Ly), grazing (Gr), total mortality (m_tot_ = Ly+Gr) and gross growth (µ_gross_) rates (d^-1^) for Phyto groups I-VI and VIII-IX. * indicates a significant difference (*p* < 0.05) of the linear regression from either: zero (µ_gross_ and Gr) or from the grazing regression (Ly); a white background indicates missing data due to either low abundance or high variation.

| Date | Phyto I (d^-1^) | | | | Phyto II (d^-1^) | | | | Phyto III (d^-1^) | | | | Phyto IV (d^-1^) | | | | Phyto V (d^-1^) | | | |
| --- | --- | --- | --- | --- | --- | --- | --- | --- | --- | --- | --- | --- | --- | --- | --- | --- | --- | --- | --- | --- |
|  | Ly | Gr | m_tot_ | µ_gross_ | Ly | Gr | m_tot_ | µ_gross_ | Ly | Gr | m_tot_ | µ_gross_ | Ly | Gr | m_tot_ | µ_gross_ | Ly | Gr | m_tot_ | µ_gross_ |
| 06/12/12 | 0.21 | 0.00 | 0.21 | 0.43 | 0.27 | 0.10 | 0.36 | 0.35* | 0.23 | 0.07 | 0.30 | 0.80 |  |  |  |  |  |  |  |  |
| 10/12/12 | 0.05 | 0.37* | 0.42 | 0.57* | 0.41* | 0.32* | 0.73* | 0.48* | 0.65* | 0.00 | 0.65* | 0.92* | 0.68* | 0.00 | 0.68* | 0.7* | 1.11* | 0.04 | 1.15* | 1.31* |
| 13/12/12 | 0.10 | 0.55* | 0.65 | 0.33* | 0.06 | 0.56* | 0.62 | 0.64* | 0.06 | 0.41* | 0.48 | 0.36* |  |  |  |  | 0.00 | 0.77* | 0.77 | 0.46* |
| 18/12/12 | 0.64* | 0.15 | 0.79* | 0.45* | 0.42* | 0.36* | 0.78* | 0.59* | 0.5* | 0.05 | 0.55* | 0.43* |  |  |  |  | 0.36 | 0.22 | 0.58 | 0.56 |
| 03/01/13 | 0.10 | 0.29* | 0.39 | 0.58* | 0.22* | 0.12 | 0.35* | 0.61* | 0.48* | 0.15 | 0.63* | 0.9* |  |  |  |  | 1.01* | 0.00 | 1.01* | 0.88* |
| 09/01/13 | 0.00 | 0.30* | 0.30* | 0.42* | 0.00 | 0.43* | 0.43* | 0.39* | 0.05 | 0.22* | 0.27 | 0.27* |  |  |  |  | 0.02 | 0.49* | 0.51* | 0.59* |
| 14/01/13 | 0.18 | 0.39* | 0.58 | 0.52* | 0.30 | 0.26* | 0.56 | 0.59* | 0.21 | 0.21* | 0.42 | 1.12* | 0.76* | 0.00 | 0.77* | 0.71* | 0.44 | 0.06 | 0.51 | 0.64* |
| 21/01/13 | 0.00 | 0.66* | 0.66 | 0.77* | 0.19 | 0.55* | 0.74 | 0.59* | 0.00 | 0.32* | 0.32 | 0.56* | 0.00 | 0.63 | 0.63 | 0.97* | 0.25 | 0.23 | 0.49 | 0.88 |
| 28/01/13 | 0.10 | 1.25* | 1.35 | 1.31* | 0.41* | 0.29* | 0.7* | 0.78* | 0.00 | 0.44* | 0.44 | 0.54 | 0.79* | 0.27* | 1.06* | 0.78* | 0.03 | 0.91* | 0.94 | 0.69* |
| 05/02/13 | 0.81* | 0.16 | 0.97* | 0.79* |  |  | 0.25* | 0.25* | 0.19 | 0.00 | 0.19 | 0.06* | 0.83* | 0.05 | 0.88* | 0.74* | 0.08 | 0.34 | 0.42 | 0.57* |
| 11/02/13 | 0.03 | 0.81 | 0.84 | 0.68* | 0.00 | 0.53* | 0.53 | 0.08* | 0.35 | 0.47* | 0.82 | 1.22* | 0.67* | 0.55* | 1.22* | 0.96* | 0.37 | 0.86* | 1.23 | 0.94* |
| 19/02/13 | 1.03* | 0.01 | 1.04* | 0.71* | 0.55* | 0.06 | 0.61* | 0.45* | 0.21* | 0.26* | 0.47* | 0.5* | 0.01 | 0.65* | 0.66 | 0.47* | 0.01 | 0.91* | 0.92 | 0.9* |
| 28/02/13 | 0.00 | 0.00 | 0.00 | 0.34 | 0.01 | 0.11 | 0.12 | 0.23 | 0.36* | 0.14 | 0.5* | 0.23* | 0.00 | 0.10 | 0.10 | 0.43 | 0.12 | 0.00 | 0.12 | 0.07 |
| 05/03/13 | 0.72* | 0.16 | 0.88* | 0.69* | 0.38* | 0.03 | 0.4* | 0.05* | 0.42* | 0.17 | 0.59* | 0.37* | 0.07 | 0.00 | 0.07 | 0.03 | 0.09 | 0.21 | 0.30 | 0.23 |
| 12/03/13 |  |  |  |  |  |  |  |  | 0.00 | 0.31* | 0.31 | 0.22 |  |  |  |  | 0.13 | 0.47 | 0.60 | 0.00 |
|  |  |  |  |  |  |  |  |  |  |  |  |  |  |  |  |  |  |  |  |  |
| Date | Phyto VI (d^-1^) | | | | Phyto VIII (d^-1^) | | | | Phyto IX (d^-1^) | | | |  |  |  |  |  |  |  |  |
|  | Ly | Gr | m_tot_ | µ_gross_ | Ly | Gr | m_tot_ | µ_gross_ | Ly | Gr | m_tot_ | µ_gross_ |  |  |  |  |  |  |  |  |
| 06/12/12 | 1.34* | 0.09* | 1.43* | 1.29* | 0.58 | 0.35 | 0.92 | 0.37 | 0.19 | 0.32 | 0.51 | 0.86 |  |  |  |  |  |  |  |  |
| 10/12/12 | 0.01 | 0.00 | 0.01 | 0.67 |  |  |  |  | 0.25 | 0.27 | 0.52 | 1.08 |  |  |  |  |  |  |  |  |
| 13/12/12 | 0.11 | 0.74 | 0.85 | 1.03 | 0.00 | 0.66* | 0.66 | 0.74 | 0.35 | 0.59* | 0.94 | 0.96* |  |  |  |  |  |  |  |  |
| 18/12/12 |  | 0.93* |  |  | 0.40* | 0.04 | 0.44* | 1.05* | 0.14 | 0.11 | 0.26 | 0.18 |  |  |  |  |  |  |  |  |
| 03/01/13 | 0.31 | 0.34* | 0.65 | 0.78* | 0.42 | 0.58* | 1.00 | 1.12* |  |  |  |  |  |  |  |  |  |  |  |  |
| 09/01/13 | 0.00 | 0.04 | 0.04 | 0.47 |  |  |  |  |  |  |  |  |  |  |  |  |  |  |  |  |
| 14/01/13 | 0.24 | 0.32* | 0.56 | 0.78* |  |  |  |  | 0.38 | 0.24 | 0.62 | 0.91* |  |  |  |  |  |  |  |  |
| 21/01/13 | 0.08 | 0.68* | 0.76 | 0.98* | 0.00 | 0.4* | 0.40 | 0.85* | 0.00 | 0.12 | 0.12 | 0.33 |  |  |  |  |  |  |  |  |
| 28/01/13 | 0.59* | 0.36* | 0.96* | 1.18* | 0.00 | 0.92 | 0.92 | 0.38 |  |  |  |  |  |  |  |  |  |  |  |  |
| 05/02/13 | 0.00 | 0.20 | 0.20 | 0.64 | 0.70 | 0.00 | 0.70 | 0.47 | 0.60 | 0.01 | 0.61 | 0.64* |  |  |  |  |  |  |  |  |
| 11/02/13 | 0.27 | 0.72 | 1.00 | 1.08 |  |  |  |  | 0.26 | 0.57* | 0.82 | 0.72* |  |  |  |  |  |  |  |  |
| 19/02/13 | 0.00 | 0.43* | 0.43 | 0.44* | 0.00 | 0.78* | 0.78 | 0.78* | 0.44* | 0.07 | 0.5* | 0.52* |  |  |  |  |  |  |  |  |
| 28/02/13 |  |  |  |  | 0.69* | 0.31 | 1.01* | 0.66* |  |  | 0.06 | 0.09 |  |  |  |  |  |  |  |  |
| 05/03/13 | 0.00 | 0.26* | 0.26 | -0.04* | 0.66* | 0.00 | 0.66* | 0.37* | 0.38* | 0.11 | 0.49* | 0.31* |  |  |  |  |  |  |  |  |
| 12/03/13 | 0.03 | 0.13 | 0.16 | -0.05 |  |  |  |  |  | 0.28* |  |  |  |  |  |  |  |  |  |  |

Table S2. Pearson correlation coefficients (r) between viral lysis (Ly) and grazing (Gr) rates, as well as significance (*p*) and number of datapoints (*n*), for all phytoplankton groups (Phyto ALL) and specifically for groups Phyto I, III and V.

^a^ Coefficients are also shown for Phyto I with one datapoint excluded, i.e. Ly and Gr are both 0.00 d^-1^ (see Fig. 2b).

| **Variables** | **r** | ***p*** | ***n*** |
| --- | --- | --- | --- |
| Phyto All | -0.427 | <0.0001 | 98 |
| Phyto I | -0.470 | 0.0901 | 14 |
| Phyto I ^a^ | -0.579 | 0.0380 | 13 |
| Phyto III | -0.600 | 0.0180 | 15 |
| Phyto V | -0.561 | 0.0369 | 14 |

Table S3. Carbon lost by viral lysis (Ly), grazing (Gr), total mortality (m_tot_ =Ly+Gr) and carbon produced by gross growth (µ_gross_) rates (µg C L^-1^ d^-1^) for Phyto groups I-VI and VIII-IX. St.st. is the standing stock in µg C L^-1^.

Note: No values indicate missing data due to either very low abundance and/or too high variation to obtain reliable estimates. For statistical analysis only sets with both lysis and grazing data were used.

| Date | Phyto I (µg C L^-1^ d^-1^) | | | | | Phyto II (µg C L^-1^ d^-1^) | | | | | Phyto III (µg C L^-1^ d^-1^) | | | | | Phyto IV (µg C L^-1^ d^-1^) | | | | | Phyto V (µg C L^-1^ d^-1^) | | | | |
| --- | --- | --- | --- | --- | --- | --- | --- | --- | --- | --- | --- | --- | --- | --- | --- | --- | --- | --- | --- | --- | --- | --- | --- | --- | --- |
|  | Ly | Gr | m_tot_ | µ_gross_ | St.st. | Ly | Gr | m_tot_ | µ_gross_ | St.st. | Ly | Gr | m_tot_ | µ_gross_ | St.st. | Ly | Gr | m_tot_ | µ_gross_ | St.st. | Ly | Gr | m_tot_ | µ_gross_ | St.st. |
| 06/12/12 | 0.06 | 0.00 | 0.06 | 0.13 | 0.26 | 0.45 | 0.16 | 0.61 | 0.59 | 1.69 | 0.59 | 0.19 | 0.78 | 2.06 | 1.98 |  |  |  |  | 0.14 |  |  |  |  | 0.36 |
| 10/12/12 | 0.00 | 0.03 | 0.03 | 0.04 | 0.07 | 0.59 | 0.45 | 1.04 | 0.69 | 1.60 | 3.08 | 0.00 | 3.08 | 4.39 | 4.13 | 0.20 | 0.00 | 0.20 | 0.20 | 0.28 | 0.64 | 0.03 | 0.66 | 0.76 | 0.53 |
| 13/12/12 | 0.01 | 0.03 | 0.04 | 0.02 | 0.06 | 0.07 | 0.66 | 0.73 | 0.75 | 1.16 | 0.26 | 1.73 | 1.99 | 1.51 | 4.43 |  |  |  |  | 0.04 | 0.00 | 0.54 | 0.54 | 0.32 | 0.82 |
| 18/12/12 | 0.05 | 0.01 | 0.06 | 0.04 | 0.10 | 0.69 | 0.58 | 1.27 | 0.96 | 1.79 | 2.37 | 0.25 | 2.62 | 2.04 | 5.04 |  |  |  |  | 0.22 | 0.53 | 0.32 | 0.85 | 0.82 | 1.47 |
| 03/01/13 | 0.00 | 0.01 | 0.02 | 0.03 | 0.04 | 0.32 | 0.17 | 0.49 | 0.87 | 1.23 | 2.94 | 0.88 | 3.82 | 5.46 | 5.29 |  |  |  |  | 1.12 | 1.74 | 0.00 | 1.74 | 1.52 | 1.83 |
| 09/01/13 | 0.00 | 0.01 | 0.01 | 0.02 | 0.04 | 0.00 | 0.16 | 0.16 | 0.14 | 0.37 | 0.15 | 0.67 | 0.82 | 0.80 | 3.02 |  |  |  |  | 0.29 | 0.01 | 0.39 | 0.40 | 0.47 | 0.76 |
| 14/01/13 | 0.02 | 0.05 | 0.07 | 0.06 | 0.12 | 0.93 | 0.83 | 1.76 | 1.85 | 3.10 | 1.37 | 1.33 | 2.70 | 7.18 | 4.43 | 3.15 | 0.02 | 3.17 | 2.92 | 4.25 | 1.48 | 0.21 | 1.69 | 2.15 | 3.12 |
| 21/01/13 | 0.00 | 0.01 | 0.01 | 0.02 | 0.02 | 0.30 | 0.89 | 1.19 | 0.95 | 1.73 | 0.00 | 0.90 | 0.90 | 1.61 | 2.52 | 0.00 | 4.97 | 4.97 | 7.62 | 6.63 | 0.87 | 0.79 | 1.66 | 3.00 | 2.79 |
| 28/01/13 | 0.00 | 0.00 | 0.00 | 0.00 | 0.00 | 0.07 | 0.05 | 0.12 | 0.14 | 0.17 | 0.00 | 0.80 | 0.80 | 0.97 | 1.73 | 4.41 | 1.53 | 5.93 | 4.38 | 6.41 | 0.04 | 1.16 | 1.20 | 0.88 | 1.44 |
| 05/02/13 | 0.00 | 0.00 | 0.00 | 0.00 | 0.01 |  |  |  |  | 0.24 | 0.52 | 0.01 | 0.53 | 0.17 | 2.95 | 1.51 | 0.09 | 1.60 | 1.34 | 1.95 | 0.22 | 1.01 | 1.23 | 1.69 | 2.72 |
| 11/02/13 | 0.00 | 0.01 | 0.01 | 0.00 | 0.01 | 0.00 | 0.10 | 0.10 | 0.01 | 0.23 | 1.88 | 2.54 | 4.42 | 6.58 | 4.39 | 3.22 | 2.64 | 5.86 | 4.61 | 5.47 | 0.86 | 2.00 | 2.85 | 2.17 | 2.67 |
| 19/02/13 | 0.01 | 0.00 | 0.01 | 0.00 | 0.01 | 0.16 | 0.02 | 0.18 | 0.13 | 0.31 | 1.28 | 1.64 | 2.93 | 3.13 | 6.15 | 0.03 | 1.46 | 1.49 | 1.05 | 2.48 | 0.05 | 3.50 | 3.55 | 3.46 | 3.88 |
| 28/02/13 | 0.00 | 0.00 | 0.00 | 0.00 | 0.00 | 0.00 | 0.02 | 0.02 | 0.03 | 0.14 | 1.83 | 0.70 | 2.53 | 1.18 | 5.81 | 0.00 | 0.03 | 0.03 | 0.14 | 0.28 | 0.25 | 0.00 | 0.25 | 0.15 | 2.11 |
| 05/03/13 | 0.00 | 0.00 | 0.01 | 0.00 | 0.01 | 0.05 | 0.00 | 0.06 | 0.01 | 0.16 | 1.54 | 0.62 | 2.16 | 1.34 | 4.09 | 0.03 | 0.00 | 0.03 | 0.01 | 0.44 | 0.10 | 0.22 | 0.33 | 0.24 | 1.12 |
| 12/03/13 |  |  |  |  | 0.01 |  |  |  |  | 0.08 | 0.00 | 1.00 | 1.00 | 0.69 | 3.36 |  |  |  |  | 0.27 | 0.11 | 0.38 | 0.49 | 0.00 | 1.08 |
|  |  |  |  |  |  |  |  |  |  |  |  |  |  |  |  |  |  |  |  |  |  |  |  |  |  |
| Date | Phyto VI (µg C L^-1^ d^-1^) | | | | | Phyto VIII (µg C L^-1^ d^-1^) | | | | | Phyto IX (µg C L^-1^ d^-1^) | | | | | Total (µg C L^-1^ d^-1^) | | | | |  |  |  |  |  |
|  | Ly | Gr | m_tot_ | µ_gross_ | St.st. | Ly | Gr | m_tot_ | µ_gross_ | St.st. | Ly | Gr | m_tot_ | µ_gross_ | St.st. | Ly | Gr | m_tot_ | µ_gross_ | St.st. |  |  |  |  |  |
| 06/12/12 | 7.84 | 0.51 | 8.35 | 7.55 | 6.25 | 6.08 | 3.66 | 9.75 | 3.94 | 13.74 | 5.80 | 9.88 | 15.68 | 26.76 | 25.84 | 20.8 | 14.4 | 35.2 | 41 | 52.9 |  |  |  |  |  |
| 10/12/12 | 0.02 | 0.00 | 0.02 | 1.15 | 1.21 |  |  |  |  | 0.67 | 7.17 | 7.87 | 15.04 | 31.08 | 21.48 | 11.7 | 8.4 | 20.1 | 38.3 | 33.1 |  |  |  |  |  |
| 13/12/12 | 0.26 | 1.67 | 1.93 | 2.35 | 2.08 | 0.00 | 2.73 | 2.73 | 3.04 | 3.96 | 45.11 | 75.92 | 121 | 123.7 | 127.6 | 45.7 | 83.3 | 129 | 131.7 | 141.3 |  |  |  |  |  |
| 18/12/12 |  |  |  |  | 19.06 | 23.21 | 2.43 | 25.64 | 61.42 | 42.42 | 33.15 | 26.18 | 59.33 | 41.47 | 240.3 | 60 | 29.8 | 89.8 | 106.7 | 312.9 |  |  |  |  |  |
| 03/01/13 | 4.48 | 4.84 | 9.32 | 11.22 | 13.50 | 2.86 | 3.95 | 6.81 | 7.65 | 6.41 |  |  |  |  | 0.78 | 12.3 | 9.9 | 22.2 | 26.7 | 31.5 |  |  |  |  |  |
| 09/01/13 | 0.00 | 0.27 | 0.27 | 3.64 | 6.12 |  |  |  |  | 10.21 |  |  |  |  | 21.02 | 0.2 | 1.5 | 1.7 | 5.1 | 43 |  |  |  |  |  |
| 14/01/13 | 10.56 | 13.90 | 24.47 | 33.82 | 39.13 |  |  |  |  | 2.39 | 4.43 | 2.86 | 7.28 | 10.74 | 10.12 | 22 | 19.2 | 41.1 | 58.7 | 67.8 |  |  |  |  |  |
| 21/01/13 | 1.25 | 10.55 | 11.79 | 15.09 | 13.84 | 0.00 | 5.46 | 5.46 | 11.55 | 10.70 | 0.00 | 0.55 | 0.55 | 1.53 | 4.20 | 2.4 | 24.1 | 26.5 | 41.4 | 42.8 |  |  |  |  |  |
| 28/01/13 | 3.93 | 2.40 | 6.32 | 7.79 | 5.91 | 0.00 | 1.78 | 1.78 | 0.73 | 2.50 |  |  |  |  | 1.87 | 8.4 | 7.7 | 16.2 | 14.9 | 20.3 |  |  |  |  |  |
| 05/02/13 | 0.00 | 3.36 | 3.36 | 10.57 | 13.16 | 1.26 | 0.00 | 1.26 | 0.83 | 2.01 | 3.89 | 0.07 | 3.96 | 4.16 | 6.38 | 7.4 | 4.5 | 11.9 | 18.8 | 30.3 |  |  |  |  |  |
| 11/02/13 | 6.23 | 16.44 | 22.67 | 24.65 | 21.78 |  |  |  |  | 1.68 | 1.25 | 2.76 | 4.01 | 3.50 | 5.14 | 13.4 | 26.5 | 39.9 | 41.5 | 41.7 |  |  |  |  |  |
| 19/02/13 | 0.00 | 18.64 | 18.64 | 19.09 | 43.45 | 0.00 | 4.29 | 4.29 | 4.28 | 5.49 | 49.91 | 7.59 | 57.50 | 58.69 | 113.3 | 51.4 | 37.1 | 88.6 | 89.8 | 176.1 |  |  |  |  |  |
| 28/02/13 |  |  |  |  | 34.64 | 1.08 | 0.49 | 1.57 | 1.04 | 1.85 |  |  |  |  | 164.7 | 3.2 | 1.2 | 4.4 | 2.5 | 209.8 |  |  |  |  |  |
| 05/03/13 | 0.00 | 4.28 | 4.28 | -0.75 | 19.44 | 2.67 | 0.00 | 2.67 | 1.49 | 4.67 | 131.1 | 36.86 | 167.9 | 107 | 372.4 | 135.5 | 42 | 177.5 | 109.4 | 403 |  |  |  |  |  |
| 12/03/13 | 0.18 | 0.70 | 0.87 | -0.27 | 5.99 |  |  |  |  | 5.0 |  |  |  |  | 510.9 | 0.3 | 2.1 | 2.4 | 0.4 | 527 |  |  |  |  |  |

**References**

1. Annett AL, Skiba M, Henley SF, Venables HJ, Meredith MP, Statham PJ, et al. Comparative roles of upwelling and glacial iron sources in Ryder Bay, coastal western Antarctic Peninsula. *Mar Chem* 2015; **176**: 21–33.
